# A Comparison of the Efficacy and Safety of Denosumab and Zoledronic Acid in Patients with Bone Metastatic Breast Cancer Receiving CDK4/6 Inhibitor Therapy

**DOI:** 10.3390/medicina61020360

**Published:** 2025-02-19

**Authors:** İrem Öner, Hicran Anık, Bediz Kurt İnci, Pınar Kubilay Tolunay, Öztürk Ateş, Ülkü Yalçıntaş Arslan, Cengiz Karaçin

**Affiliations:** Departmant of Medical Oncology, Dr. Abdurrahman Yurtaslan Ankara Oncology Research and Training Hospital, 06200 Ankara, Turkey; hicrancilanik@gmail.com (H.A.); bedizkurt@gmail.com (B.K.İ.); pinar_kubilay@hotmail.com (P.K.T.); dr.ozturkates@gmail.com (Ö.A.); ulkuarslan63@gmail.com (Ü.Y.A.); cengizkaracin@yahoo.com (C.K.)

**Keywords:** cdk4/6 inhibitor, ribociclib, palbociclib, denosumab, RANKL inhibitor, zoledronic acid, skeletal-related event, breast cancer, bone metastasis

## Abstract

*Background and Objectives*: Bone metastases in patients can cause significant quality-of-life declines due to skeletal-related events (SREs). SRE is defined as the occurrence of radiotherapy for bone pain, pathologic fracture, bone surgery, spinal cord compression, or hypercalcemia. Bone-modifying agents (BMAs), such as denosumab and zoledronic acid, are crucial in reducing the frequency and severity of SREs. The inhibition of cyclin-dependent kinase 4/6 (CDK4/6) inhibitors has emerged as the standard treatment for hormone receptor-positive metastatic breast cancer, demonstrating significant improvements in survival outcomes. This study aims to compare the effectiveness of denosumab and zoledronic acid in preventing SRE in patients receiving CDK4/6 inhibitors with endocrine therapy. *Materials and Methods*: This retrospective study included 328 patients diagnosed with bone metastatic breast cancer receiving first-line CDK4/6 inhibitor therapy (palbociclib or ribociclib). Patients were assigned to receive either subcutaneous denosumab or intravenous zoledronic acid every 4 weeks. Time to the first skeletal-related event post bone-modifying agent initiation, SRE incidence, and the safety data were evaluated. The data were analyzed using independent samples t-tests, chi-square tests, and Kaplan–Meier methods for time-to-event data. *Results*: In the denosumab group, the median time to the first skeletal-related event was significantly longer than in the zoledronic acid group (44.55 months and 29.16 months, respectively). Denosumab treatment was associated with a statistically significant reduction in the risk of developing the first SRE after bone-modifying agent initiation compared to zoledronic acid (HR: 0.56, *p* = 0.001). Additionally, ECOG PS and the number of metastatic bone sites were identified as independent prognostic factors for time to the first SRE. The safety profile was consistent with previous studies reported in the literature. *Conclusions*: Our study demonstrated that when used with CDK4/6 inhibitors, denosumab is associated with a delay in SREs and a lower SRE incidence than zoledronic acid in patients with bone metastases. These findings support the efficacy of denosumab in preventing SREs and suggest that CDK4/6 inhibitors may have distinct effects on the bone microenvironment, particularly when combined with denosumab.

## 1. Introduction

Women are most frequently diagnosed with breast cancer globally, which also represents the leading cause of cancer deaths among them [1]. Advances in breast cancer treatments have led to increased survival rates for individuals with metastatic disease [2]. Bone metastasis is considered an incurable disease, and treatments are typically palliative [3]. Consequently, bone metastases represent a significant health concern, and their prevalence is expected to rise further in the future due to the increasing incidence of cancer in an aging population [4].

Bone metastases present a significant clinical challenge in the context of advanced breast cancer, with reported incidence rates ranging from 65% to 75% [5]. Bone metastases can lead to a range of skeletal-related events (SREs), significantly impacting patient morbidity and potentially causing complications such as severe pain, skeletal instability, and metabolic disturbances. These events can significantly compromise a patient’s overall well-being and quality of life [6].

Bone-modifying agents (BMAs) are critical in mitigating the incidence and severity of SREs in patients with bone metastases [7]. Bisphosphonates’ ultimate effect is a reduction in bone resorption, achieved through the inhibition of osteoclast differentiation, induction of apoptosis, suppression of function, and prevention of bone surface attachment [8]. Denosumab is a monoclonal antibody targeting RANKL (receptor activator of nuclear factor-kappa B ligand). RANKL inhibition prevents osteoclast differentiation and activation. Denosumab and zoledronic acid differ in their mechanisms of action, pharmacokinetic properties, administration methods, and cost-effectiveness [8,9].

The inhibition of cyclin-dependent kinase 4/6 (CDK4/6) activity blocks cell cycle progression, leading to G1 arrest and the inhibition of tumor cell proliferation [10]. In women with hormone receptor (HR)-positive, human epidermal growth factor receptor 2 (HER2)-negative advanced breast cancer, pivotal clinical trials have demonstrated that CDK4/6 inhibitors, including ribociclib, palbociclib, and abemaciclib, significantly improve progression-free survival [11,12,13]. Studies investigating ribociclib and abemaciclib have revealed overall survival benefits in this patient population [14,15]. Thus, CDK4/6 inhibitors plus endocrine therapy (ET) are utilized as the standard treatment for this patient population.

Some studies with CDK4/6 inhibitors suggest the potential to slow disease progression or control tumor growth in bone metastasis sites, specifically within the subgroup of patients with bone metastases from the overall patient population [16,17,18]. However, data on these drugs’ direct and indirect effects on bone are limited. One study investigating the effects of CDK4/6 inhibitors on the breast cancer bone microenvironment has shown effects on osteoclast differentiation and bone resorption [19]. Several in vivo studies have also demonstrated the effect of palbociclib on bone metastases and the efficacy of the RANK pathway and CDK4/6 inhibition in luminal breast cancer [20,21].

Despite growing evidence of the potential of CDK4/6 inhibitors to slow bone progression in breast cancer patients with bone metastases, the effects of these drugs on the bone microenvironment are not fully understood, and the clinical implications of these effects remain unclear. Furthermore, the type of bone-modifying agent used (denosumab or zoledronic acid) during CDK4/6 inhibitor treatment may differ in efficacy. This study aims to compare the effectiveness and safety profiles of denosumab and zoledronic acid in controlling bone resorption in patients diagnosed with bone metastatic breast cancer and receiving CDK4/6 inhibitor treatment, thereby providing important information for determining the most appropriate clinical approach and optimizing patient care.

## 2. Materials and Methods

Patients diagnosed with bone metastatic breast cancer who received first-line CDK4/6 inhibitor therapy (palbociclib or ribociclib) plus endocrine therapy between October 2017 and January 2024 at our center were identified. The patients consisted of those with de novo bone metastatic disease or those with early-stage breast cancer who had received prior treatment (adjuvant or neoadjuvant) and developed new bone metastases and who received denosumab or zoledronic acid as the initial therapy for metastatic disease. Patients who received denosumab or zoledronic acid, along with a prior line of endocrine therapy, were excluded from the analysis.

Before initiating systemic and antiresorptive therapy, patients had radiologically confirmed bone metastases (via an X-ray, computed tomography, positron emission tomography, or magnetic resonance imaging) with at least one verified lesion. They also had an Eastern Cooperative Oncology Group performance status (ECOG PS) of 2 or less, adequate organ function, a baseline creatinine clearance of 30 mL/min or greater, and a history of treatment with a BMA for at least 3 months.

Patients with a tissue immunohistochemistry (IHC) score of 10% or higher for estrogen receptor expression were included, following established CDK4/6 inhibitor approval and use criteria. HER2 status was categorized as HER2-zero for those with IHC 0 and HER2-low for those with IHC 1+ or IHC 2+ who were negative for in situ hybridization. Patients were categorized into three groups based on the number of bone metastases: less than 2, 2–5, and more than 5. An SRE was defined as the need for radiation therapy (RT) to bone (for pain or impending fracture), pathologic bone fracture, surgery to bone, spinal cord compression, or hypercalcemia.

In addition to systemic therapy, patients were treated with either 120 mg denosumab subcutaneously every 28 days or 4 mg zoledronic acid intravenously every 28 days for a minimum of three months. Patients previously receiving bisphosphonate treatment for bone metastases who had undergone unresolved dental/oral surgery or had a pre-treatment baseline creatinine clearance of less than 30 mL/min were excluded from the study. Patient demographic information, metastatic disease characteristics, treatment timelines, and start dates of denosumab or zoledronic acid used with CDK4/6 inhibitors were collected from electronic patient records. The necessary ethical approvals for this study were obtained following institutional guidelines.

Adverse events were examined in two categories: common and specific. Common adverse events included nausea, fatigue, back pain, arthralgia, dyspnea, diarrhea, anemia, leukopenia, neutropenia and tromboctopenia. As these events can be associated with both BMAs and CDK4/6 inhibitors, they were analyzed considering the treatment regimen as a whole. Therefore, common adverse events were assessed to encompass all components of the treatment regimen. Adverse events associated with a particular component of the treatment were reported separately under specific adverse events. Specific adverse events were defined as hypocalcemia, medication-related osteonecrosis (MRONJ) of the jaw, and renal impairment. The incidence of MRONJ was evaluated, and this term was used to describe cases of osteonecrosis of the jaw associated with antiresorptive therapy. No prophylactic measures were implemented for patients prior to the initiation of osteoclast inhibitor therapy to mitigate the risk of hypocalcemia. However, patients who developed hypocalcemia as an adverse effect following BMA treatment received therapeutic oral calcium supplementation.

Descriptive statistics were presented as frequencies (percentages) for categorical variables and mean ± standard deviation or median (interquartile range) for continuous variables. The two study groups were compared using the independent-samples t-test for continuous variables and the Chi-square or Fisher’s exact test for categorical variables, as appropriate. The time to the first post-BMA SRE was defined as the duration from initiating BMA treatment to the occurrence of the first SRE. The time-to-event data were analyzed using the Kaplan–Meier method, and the Log-rank test was employed to compare the time to the first post-BMA SRE between the denosumab and zoledronic acid groups. All statistical tests were two-sided, and a *p*-value of less than 0.05 was considered statistically significant. Statistical analyses were performed using IBM SPSS Statistics for Windows, Version 25.0.

## 3. Results

A total of 328 patients were included in the study, with 179 (54.6%) in the denosumab group and 149 (45.4%) in the zoledronic acid group. The median age of the patients was 55 years. The treatment groups were generally similar regarding baseline characteristics (Table 1).

The median follow-up duration was 26.97 months (95% CI: 23.15–30.80) for the denosumab group and 24.68 months (95% CI: 20.36–29.00) for the zoledronic acid group, with no significant difference between the two (*p* = 0.217). During the follow-up period, 62 patients (34.6%) receiving denosumab did develop an SRE compared to 66 patients (44.3%) receiving zoledronic acid (*p* = 0.074). The distribution of the types of the first post-BMA SRE observed in the denosumab and zoledronic acid groups is detailed in Supplementary File S1 (Table S1).

**Figure 1:**
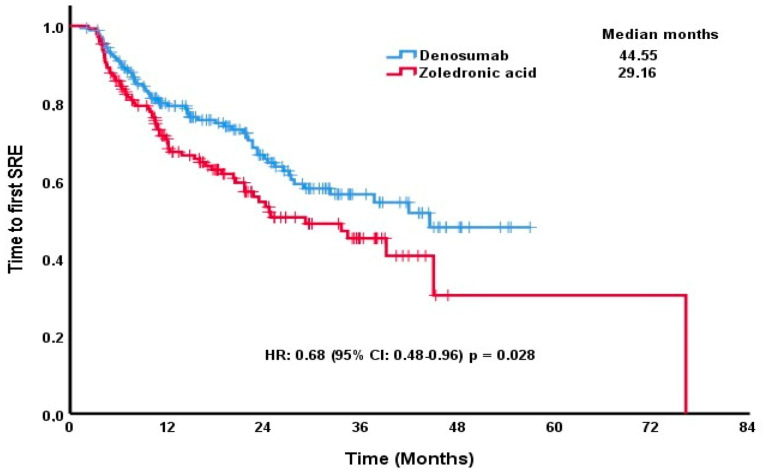
Kaplan–Meier estimates of time to the first post-BMA SRE by treatment group.

The median time to the first post-BMA SRE was significantly longer in the denosumab group compared to the zoledronic acid group: 44.55 months (95% CI: not reached (NR)) versus 29.16 months (95% CI: 18.55–39.78), respectively (*p* = 0.028) (Figure 1).

The time to the first SRE post BMA was 44.55 months in patients with an ECOG performance status of 0 compared to 27.37 months (95% CI: 23.08–31.66) in those with an ECOG score of 1. Patients with an ECOG score of 2 experienced a median time to SRE of 17.54 months (95% CI: 2.13–32.96). This difference was statistically significant (*p* = 0.001). When stratified by the number of metastatic bone lesions, the time to the first SRE post BMA was not estimable for patients with <2 lesions, while it was found to be 39.17 months (95% CI: 29.61–48.73) for those with 2–5 lesions. Patients with more than five metastatic bone lesions exhibited a significantly shorter time of 27.76 months (95% CI: 17.70–37.82) (*p* = 0.007) (Table 2). The correlation between the number of bone metastases and the delay in the need for radiotherapy was evaluated in patients treated with denosumab and zoledronic acid. In patients with two or fewer metastatic bone lesions, the median time to RT was not reached in either treatment group, and there was no statistically significant difference between the groups (*p* = 0.412). In patients with 2–5 lesions, the median time to RT was 34.40 months (95% CI: 21.72–47.08) in the zoledronic acid group and 44.55 months (95% CI: 19.72–69.38) in the denosumab group; however, this difference was not statistically significant (*p* = 0.893). In patients with more than five metastatic bone lesions, the median time to RT was 21.62 months (95% CI: 14.08–29.16) in the zoledronic acid group and 41.96 months (95% CI: NE) in the denosumab group, with a statistically significant difference observed between the groups (*p* = 0.012). Further analysis revealed no statistically significant differences in time to the first post-BMA SRE between patient groups stratified by factors such as median age, menopausal status, HER2 status, the presence of visceral metastases, the type of CDK4/6 inhibitor used (ribociclib or palbociclib), the type of endocrine therapy used (letrozole or fulvestrant), and the type of SRE.

Cox regression analysis demonstrated that denosumab significantly prolonged the time to the first SRE after BMA compared to zoledronic acid (HR: 0.56, 95% CI: 0.39–0.79, *p* = 0.001). A strong association was observed between ECOG performance status and time to the first SRE after BMA. Patients with an ECOG score of 1 had an HR of 2.67 (95% CI: 1.55–4.59) (*p* < 0.001), while those with an ECOG score of 2 had an HR of 4.31 (95% CI: 2.29–8.11) (*p* < 0.001). Regarding the number of metastatic bone lesions, the HR was 4.69 (95% CI: 1.43–15.40) (*p* = 0.011) for patients with 2–5 lesions and 6.14 (95% CI: 1.91–19.76) (*p* = 0.002) for those with >5 lesions (Table 3).

Adverse events were observed in most patients, with 95.5% of those receiving denosumab and 97.3% of those receiving zoledronic acid experiencing at least one adverse event. Table 4 summarizes the most common and specific adverse events. Anemia was reported significantly less frequently in the denosumab group compared to the zoledronic acid group (20.1% vs. 30.2%, *p* = 0.035). However, the two treatment groups had no significant differences in the incidence of other common adverse events. Among the specific adverse events, hypocalcemia, MRONJ, and renal impairment were the most frequently observed. Hypocalcemia was reported significantly more often in the denosumab group (15.1%) compared to the zoledronic acid group (7.4%, *p* = 0.030). Conversely, renal impairment was observed significantly more frequently in the zoledronic acid group (9.4%) than in the denosumab group (3.4%, *p* = 0.023). Notably, there was no significant difference in the incidence of MRONJ between the two treatment groups (Table 4).

## 4. Discussion

Our study demonstrated that initiating BMA therapy with denosumab significantly reduced the risk of the first skeletal-related event compared to zoledronic acid in patients receiving first-line CDK4/6 inhibitor therapy for bone metastatic breast cancer. The median time to the first SRE after BMA initiation was 44.55 months in the denosumab group and 29.16 months in the zoledronic acid group (*p* = 0.001; HR: 0.56). Furthermore, denosumab reduced the incidence of the first SRE compared to zoledronic acid. The SRE incidence was 34.6% in the denosumab group and 44.3% in the zoledronic acid group.

These findings are consistent with previous randomized controlled trials demonstrating the superior efficacy of denosumab compared to zoledronic acid in preventing SREs in patients with advanced breast cancer and bone metastases. In a Phase III study by Stopeck et al. involving 2046 patients, the SRE incidence over a 34-month follow-up period was 30.7% in the denosumab group and 36.5% in the zoledronic acid group. The median time to the first SRE was not reached in the denosumab group, while it was calculated as 26.4 months in the zoledronic acid group (HR: 0.82; *p* < 0.001) [22]. In another Phase III trial, one or more SREs developed in 36% of the denosumab group and 44% of the zoledronic acid group (*p* = 0.021). In this study, the median time to the first SRE was not reached in the denosumab group, while it was 25.2 months in the zoledronic acid group [7].

However, it is essential to acknowledge the limitations of the existing literature. Many studies comparing denosumab and zoledronic acid were conducted before the widespread use of CDK4/6 inhibitors [7,22,23]. Additionally, many studies did not specify the systemic therapies received by participants [24,25,26]. This lack of detail regarding contemporary treatment regimens limits the generalizability of these findings to current clinical practice.

Multiple meta-analyses have demonstrated the superiority of denosumab over zoledronic acid in delaying skeletal-related events in patients with metastatic breast cancer [27,28]. This superiority is supported by meta-analyses showing that denosumab significantly delays the onset of the first SRE (OR: 0.82; *p* < 0.0001; HR: 0.86; *p* < 0.001) [27,29]. In a pooled analysis of three pivotal randomized Phase III trials, the median time to the first SRE was 27.66 months in the denosumab group and 19.45 months in the zoledronic acid group (HR: 0.83; *p* < 0.001) [30]. Ten-year long-term data also confirmed the sustained efficacy of denosumab in reducing the risk of the first SRE and significantly prolonging the time to this event (HR: 0.82; *p* < 0.001) [28]. However, these studies included not only breast cancer patients but also patients with prostate cancer, other solid tumors, and multiple myeloma. Another study in patients with solid tumors reported a median time to the first SRE of 20.6 months in the denosumab group and 16.3 months in the zoledronic acid group (HR: 0.81; *p* = 0.017) [23].

A fundamental limitation of many previous studies investigating SREs is the inherent heterogeneity of their patient populations. These studies often encompass a wide range of cancer types and lack standardized systemic therapies, making it challenging to draw definitive conclusions about the comparative efficacy of denosumab and zoledronic acid in specific clinical contexts. In contrast, our study’s strength lies in its homogenous patient population, specifically focusing on individuals with breast cancer receiving CDK4/6 inhibitor therapy in combination with endocrine therapy. This focused approach enhances the relevance and applicability of our findings to contemporary clinical practice.

Cdk6, a G1 cell cycle factor, was first shown to play a critical role in RANKL-mediated osteoclast differentiation in 2004 [31]. Recent research focusing on the effects of CDK4/6 inhibitors on the bone microenvironment in breast cancer has revealed the complex role of these agents in osteoclast activity. One study demonstrated that CDK4/6 inhibitors suppress osteoclast differentiation and the expression of bone resorption markers [19]. Another study reported that palbociclib reduces bone metastasis formation and inhibits bone tumor growth. However, it has been observed that tumor growth resumes, and the efficacy of CDK4/6 inhibitors diminishes upon treatment discontinuation. This suggests that dynamic changes within the bone microenvironment can influence treatment response [20]. An in vivo study investigating the RANK pathway and CDK4/6 inhibition indicated that RANK pathway inhibition can modulate immune responses, thereby influencing the tumor microenvironment and potentially synergizing with the antitumor activity of CDK4/6 inhibitors [21]. These findings suggest that the effects of CDK4/6 inhibitors on the bone microenvironment may interact differently with bone-targeted agents like denosumab or zoledronic acid, depending on the biological effects of the CDK4/6 inhibitors. However, the effects of CDK4/6 inhibitors on the osteoclast–osteoblast balance within the bone microenvironment and their distinct roles in the efficacy of bone-targeted therapies have not been fully elucidated [19]. Therefore, a better understanding of the impact of CDK4/6 inhibitors on the bone microenvironment is crucial for optimizing bone metastasis treatments. In our study, the RANKL inhibitor denosumab, used in conjunction with CDK4/6 inhibitors, was observed to delay SREs following bone metastasis and have a lower SRE incidence compared to zoledronic acid; this result suggests a clinical manifestation of the aforementioned mechanisms. However, this hypothesis warrants validation through prospective, randomized controlled trials, particularly in similar patient populations utilizing CDK4/6 inhibitors.

Our study identified the efficacy of ECOG PS and the number of metastatic bone lesions, in addition to the superiority of denosumab in the time to the first SRE after post BMA. This time was considerably lower with ECOG PS 0 (44.55 months) compared to those with a score of 1 (27.37 months) and 2 (17.54 months) (*p* = 0.001). Furthermore, ECOG PS status was a strong prognostic factor in multivariate analysis. While the number of studies evaluating the relationship between ECOG PS and SRE is limited, a study investigating risk factors for an SRE in patients with bone metastases from breast cancer treated with zoledronic acid found a hazard ratio of 2.08 (95% CI: 1.36–3.18) (*p* = 0.001) for ECOG PS 1 compared to ECOG PS 0 and an HR of 2.84 (95% CI: 1.58–5.11) (*p* < 0.001) for ECOG PS 2 compared to ECOG PS 0 in univariate analysis. In multivariate analysis, the HR for ECOG PS > 0 was 1.75 (95% CI: 1.15–2.66) (*p* = 0.008) [32]. Similar to our study, this study demonstrates a statistically significant relationship between ECOG performance status and SRE risk. Another study conducted in Japan also showed that high performance status is associated with an increased risk of an SRE. However, this association was not statistically significant in multivariate analysis [33].

While some studies on SREs have specified the number of patients with two or more bone metastases, the impact on SREs has not been evaluated [22,34]. A post hoc analysis of a study reporting 10-year follow-up data on denosumab showed that denosumab significantly reduced the risk of developing SREs compared to zoledronic acid, particularly in patients stratified by the number of metastases (<2 vs. ≥2 metastases) [28]. However, our study evaluated metastatic bone lesions in more detail. The time to the first SRE post BMA was not estimable in patients with less than 2 metastatic bone lesions, while it was 39.17 months in those with 2–5 lesions and 27.76 months in those with more than 5 lesions (*p* = 0.007). Furthermore, the number of metastatic bone lesions was an independent prognostic factor for SREs. These findings provide important guidance for clinical practice and offer valuable insights into the role of the number of metastatic bone lesions in the development of SREs, which can inform clinical decisions. These results should be considered when determining treatment and follow-up strategies. In our study, we evaluated the relationship between the number of bone metastases and the time to radiotherapy. Our findings suggest that increasing metastatic bone lesions accelerates the need for RT, but this effect may vary depending on the treatment group.

In patients with two or fewer metastatic bone lesions, no significant difference was observed in the time to RT between the denosumab and zoledronic acid treatment groups. This suggests that both agents have similar efficacy in this patient population. Although the median time to RT was longer in the denosumab group for patients with two to five metastatic lesions, this difference was not statistically significant. This result does not demonstrate a significant advantage of one agent over another and should be confirmed with studies in larger patient populations. In patients with more than five metastatic bone lesions, the time to RT was significantly longer with denosumab treatment compared to zoledronic acid treatment. This finding suggests that denosumab may be more effective in preventing skeletal-related events and delaying the need for RT in patients with many bone metastases.

Our study indicates that the number of metastatic bone lesions is a determining factor in the need for radiotherapy. Denosumab may provide a more sustained benefit, especially in patients with extensive bone metastases. However, larger-scale, prospective studies are needed to translate these findings more robustly into clinical practice.

Despite denosumab’s superiority in delaying skeletal-related events, its impact on pathological fractures remains less clear. While a meta-analysis reported denosumab as statistically superior to zoledronic acid in preventing pathological fractures across all tumor types (OR: 0.86; *p* = 0.04), this advantage did not hold for tumors of endodermal origin, breast cancer, or prostate cancer (OR: 0.85; *p* = 0.13) [25]. Similarly, in our current study, no significant difference was found between denosumab and zoledronic acid in preventing pathological fractures (OR: 1.75; *p* = 0.187). Other meta-analyses corroborate this finding (OR: 0.93; *p* = 0.0; OR: 0.78; *p* = 0.54) [35,36]. However, a meta-analysis encompassing 10,192 patients demonstrated that denosumab significantly reduced the risk of pathological fractures compared to a placebo (OR: 0.50) [37]. Furthermore, a study by Lipton et al. showed denosumab’s superiority over zoledronic acid in reducing the risk of pathological fractures and the need for RT in solid tumors, including breast cancer [30].

The data regarding denosumab’s effect on the need for RT are also conflicting. While one meta-analysis indicates that denosumab significantly reduces the need for RT compared to a placebo (OR: 0.51) [37], zoledronic acid has been found ineffective in this regard in breast cancer patients [38]. Another meta-analysis reported similar outcomes for denosumab and zoledronic acid concerning bone-directed RT for SREs (OR: 0.72; *p* = 0.13) [36]. However, some studies suggest a potential benefit of denosumab in delaying or reducing the need for RT. For instance, one study showed that denosumab significantly prolonged the initiation of bone-directed RT compared to zoledronic acid (HR: 0.74; *p* = 0.012) [7]. Our current study also demonstrated that denosumab significantly extended the time to bone-directed RT compared to zoledronic acid (OR: 1.78; *p* = 0.038).

Due to inconsistencies in the literature, further research is needed to clarify the role of denosumab in preventing pathological fractures and reducing the need for RT. Factors such as tumor type, metastatic burden, and patient characteristics can influence treatment outcomes and should be considered in future investigations.

Both denosumab and zoledronic acid demonstrated a relatively balanced adverse effect profile. Frequently reported adverse effects in both treatment groups aligned with those commonly reported in the literature [22,34,39]. However, it is not appropriate to solely attribute these common side effects to BMAs, as side effects such as anemia, nausea, fatigue, back pain, arthralgia, and dyspnea are also frequently observed in CDK 4/6 inhibitor treatments. In addition to this, the observed rates of anemia in our study (denosumab: 20%, zoledronic acid: 30.2%, *p* = 0.035) were consistent with findings from previous research. For instance, Stopec et al. reported anemia rates of 18.8% and 22.9% for denosumab and zoledronic acid, respectively [22]. Similarly, Henry et al. observed anemia in 27.6% of the denosumab group and 32.6% of the zoledronic acid group (*p* = 0.03) [23].

Research suggests that denosumab may offer a more favorable safety profile than zoledronic acid concerning renal failure rates. Several studies have reported lower renal failure rates among patients receiving denosumab compared to those receiving zoledronic acid. One study found a renal failure rate of 0.2% in the denosumab arm compared to 2.5% in the zoledronic acid arm [22]. Another study reported a statistically significant difference in renal failure rates, with 4.9% for denosumab and 8.5% for zoledronic acid (*p* = 0.001) [7]. Other studies have documented lower renal failure rates in denosumab groups (e.g., 7% vs. 10%, 10.9% vs. 8.3%) [23,39]. Our findings support this trend, revealing a renal failure rate of 3.4% in the denosumab group and 9.4% in the zoledronic acid group (*p* = 0.023).

While denosumab appears to be associated with a lower risk of renal failure, it is essential to consider the potential for hypocalcemia. Our study observed a higher rate of hypocalcemia in patients receiving denosumab (15.1%) than those receiving zoledronic acid (7.4%, *p* = 0.030). This finding is consistent with previous research. Stopec et al. reported hypocalcemia rates of 5.5% and 3.4% for denosumab and zoledronic acid, respectively [22], while another study reported 3.1% and 1.3% [30]. Consistent with numerous studies, our study found no significant difference in MRONJ rates between the denosumab and zoledronic acid groups [23,30,39]. This suggests that both treatments carry a similar risk for this adverse effect. An integrated analysis of three registry studies examining breast, prostate, and other solid tumors found the risk of MRONJ to be 1.9% with denosumab and 1.3% with zoledronic acid, a difference that was not statistically significant (*p* = 0.08) [40]. A meta-analysis of five randomized trials showed a higher risk of MRONJ in the denosumab group (1.7%) compared to the zoledronic acid group (1.1%), but this difference was not statistically significant [41]. The incidence of MRONJ increases with treatment duration, particularly beyond four years [42]. An analysis of three Phase III registry studies reported an MRONJ incidence of 1.1% within the first year and 4.1% thereafter in patients receiving denosumab [40].

Our study has certain limitations. The single-center, retrospective design may introduce selection bias and limit the generalizability of the findings to broader populations. The relatively small sample size restricts the study’s generalizability and statistical power. The exclusion of patients receiving zoledronic acid at three-month intervals may affect the results, limiting a comprehensive evaluation of zoledronic acid’s efficacy in this specific patient subgroup. Furthermore, physician and patient preferences regarding denosumab or zoledronic acid represent another limitation. Clinical decisions are often based on factors such as the extent of bone metastases, prior treatment responses, renal function, treatment tolerance, and the patient’s overall health status. Individual patient preferences and treatment responses may also influence the choice between denosumab and zoledronic acid. These factors should be considered when interpreting the study’s results and evaluating its limitations. Despite these limitations, our study provides valuable insights into the efficacy of denosumab and zoledronic acid in preventing SREs in patients with stage 4 breast cancer receiving CDK4/6 inhibitors.

## 5. Conclusions

Our study observed that the RANKL inhibitor denosumab, when used with CDK4/6 inhibitors, delays SREs associated with bone metastases and is associated with a lower SRE incidence than zoledronic acid. Furthermore, the type of osteoclast inhibitor used, the patient’s ECOG performance status, and the number of metastatic bone lesions were identified as independent prognostic factors for the time to the first SRE after BMA. Our findings support the efficacy of denosumab in preventing SREs in patients with bone metastases. They also suggest CDK4/6 inhibitors may have varying effects on the bone microenvironment, particularly when combined with bone-targeting agents such as denosumab. This observation may represent a clinical manifestation of mechanisms proposed in previous studies. However, a better understanding of the effects of CDK4/6 inhibitors on osteoclast–osteoblast balance and their role in treating bone metastases is needed. Prospective, randomized controlled trials are needed to confirm this hypothesis, particularly in patients using CDK4/6 inhibitors.

## Figures and Tables

**Table 1:** Baseline demographic and clinical characteristics.

Total (n = 328)		Denosumab n = 179 (54.6%)	Zoledronic Acid n = 149 (45.4%)	*p*-Value
Median age (years)				0.607
<55 y	168 (51.2%)	94 (52.5%)	74 (49.7%)	
≥55 y	160 (48.8%)	85 (47.5%)	75 (50.3%)	
Menopausal status				0.094
Pre/perimenopause	133 (40.5%)	80 (44.7%)	53 (35.6%)	
Postmenopause	195 (59.5%)	99 (55.3%)	96 (64.4%)	
ECOG PS				0.484
Score 0	80 (24.4%)	39 (21.8%)	41 (27.5%)	
Score 1	191 (58.2%)	108 (60.3%)	83 (55.7%)	
Score 2	57 (17.4%)	32 (17.9%)	25 (16.8%)	
Comorbidity				0.458
Yes	143 (44.0%)	81 (46.0%)	62 (41.6%)	
No	181 (55.7%)	94 (53.4%)	87 (58.4%)	
HER2 status				0.209
Zero	188 (57.3%)	97 (54.2%)	91 (61.1%)	
Low	140 (42.7%)	82 (45.8%)	58 (38.9%)	
Visceral metastasis				0.108
Yes	134 (40.9%)	66 (36.9%)	68 (45.6%)	
No	194 (59.1%)	113 (63.1%)	81 (54.4%)	
CDK4/6 inhibitors				0.307
Ribociclib	197 (60.1%)	103 (57.5%)	94 (63.1%)	
Palbociclib	131 (39.9%)	76 (42.5%)	55 (36.9%)	
Endocrine agent				0.523
Letrozole	226 (68.9%)	126 (70.4%)	100 (67.1%)	
Fulvestrant	102 (31.1%)	53 (29.6%)	49 (32.9%)	
Number of metastatic bone lesions				0.755
<2	19 (5.8%)	9 (5.0%)	10 (6.7%)	
2–5	99 (30.2%)	56 (31.3%)	43 (28.9%)	
>5	210 (64.0%)	114 (63.7%)	96 (64.4%)	

ECOG PS: Eastern Cooperative Oncology Group performance status; HER2: human epidermal growth factor receptor 2; CDK4/6: cyclin-dependent kinase 4/6.

**Table 2:** Time to first skeletal-related events in patients given CDK4/6 Inhibitors.

Time to First Post-BMA SRE Median (95%CI)		*p*-Value
Median age (years)		0.725
<55 y	37.68 (21.59–53.78)	
≥55 y	39.17 (27.81–50.53)	
Menopausal status		0.863
Pre/perimenopause	76.29 (NE)	
Postmenopause	37.68 (26.95–48.41)	
ECOG PS		0.001 *
Score 0	44.55 (NE)	
Score 1	27.37 (23.08–31.66)	
Score 2	17.54 (2.13–32.96)	
HER2 status		0.909
Zero	41.96 (27.70–56.21)	
Low	37.68 (26.46–48.91)	
Visceral metastasis		0.578
Yes	32.23 (19.26–45.20)	
No	76.29 (NE)	
CDK4/6 inhibitors		0.427
Ribociclib	33.59 (22.24–44.95)	
Palbociclib	44.55 (23.51–65.60)	
Endocrine agent		0.486
Letrozole	37.68 (29.98–45.39)	
Fulvestrant	41.96 (25.56–58.35)	
Osteoclast inhibitor agent		0.028 *
Denosumab	44.55 (NE)	
Zoledronic acid	29.16 (18.55–39.78)	
Number of metastatic bone lesions		0.007 *
<2	NE (NE)	
2–5	39.17 (29.61–48.73)	
>5	27.76 (17.70–37.82)	
Type of first post-BMA SRE		0.416
RT to bone	11.07 (9.29–12.85)	
Fracture	9.69 (5.38–14.0)	
Surgery to bone	14.55 (8.18–20.93)	
Cord compression	6.44 (5.64–7.24)	
Hypercalcemia	3.65 (0.00–7.55)	

SRE: skeletal-related event; BMA: bone-modifying agent; ECOG PS: Eastern Cooperative Oncology Group performance status; HER2: human epidermal growth factor receptor 2; CDK4/6: cyclin-dependent kinase 4/6; RT: radiotherapy; NE: not-estimable; * significant.

**Table 3:** Cox regression analysis.

HR (%95 CI)		*p* Value
Osteoclast inhibitor agent		
Zoledronic acid	Ref	
Denosumab	0.56 (0.39–0.79)	0.001 *
ECOG PS		
Score 0	Ref	
Score 1	2.67 (1.55–4.59)	<0.001 *
Score 2	4.31 (2.29–8.11)	<0.001 *
Number of metastatic bone lesions		
<2	Ref	
2–5	4.69 (1.43–15.40)	0.011 *
>5	6.14 (1.91–19.76)	0.002 *

HR: hazard ratio; CI: confidence interval; Ref: reference; ECOG PS: Eastern Cooperative Oncology Group performance status: * Significant.

**Table 4:** Adverse events.

Denosumab (n = 179)		Zoledronic Acid (n = 149)	*p*-Value
Any adverse events	171 (95.5%)	145 (97.3%)	
Common Adverse Reactions			
Nausea	53 (30.6%)	47 (33.8%)	0.550
Fatigue	46 (27.4%)	40 (29.4%)	0.696
Back pain	36 (21.3%)	22 (16.8%)	0.327
Arthralgia	28 (15.6%)	31 (20.8%)	0.225
Dyspnea	36 (20.1%)	38 (25.5%)	0.245
Diarrhea	45 (26.0%)	32 (21.5%)	0.341
Anemia	36 (20.1%)	45 (30.2%)	0.035 *
Leukopenia	71 (39.7%)	48 (32.2%)	0.162
Neutropenia	141 (78.8%)	113 (75.8%)	0.527
Thrombocytopenia	21 (12.1%)	17 (11.7%)	0.925
Specific Adverse Reactions			
Hypocalcemia	27 (15.1%)	11 (7.4%)	0.030 *
MRONJ	12 (6.7%)	6 (4.0%)	0.289
Renal insufficiency	6 (3.4%)	14 (9.4%)	0.023 *

MRONJ: medication-related osteonecrosis of the jaw, * significant.

## Data Availability

In accordance with institutional policies and to protect patient confidentiality, the datasets used in this study are not available for sharing.

## Supplementary Materials

The following supporting information can be downloaded at: https://www.mdpi.com/article/10.3390/medicina61020360/s1, Table S1: The first SRE after the initiation of BMA.

## Abbreviations

SRE	Skeletal-related event
BMA	Bone-modifying agent
RANKL	Receptor activator of NF-kappaB ligand
HR	Hormone receptor
HER2	Human epidermal growth factor receptor 2
Cdk4/6	Cyclin-dependent kinase 4/6
ECOG PS	Eastern Cooperative Oncology Group performance status
IHC	Immunohistochemistry
RT	Radiotherapy
NA	Not applicable
NR	Not reached
NE	Not-estimated
HR	Hazard ratio
CI	Confidence interval
OR	Odds ratio
