# Supplementary material for: A Comparison of the Efficacy and Safety of Denosumab and Zoledronic Acid in Patients with Bone Metastatic Breast Cancer Receiving CDK4/6 Inhibitor Therapy
Source: Medicina (Kaunas). 2025 Feb 19;61(2):360. doi: 10.3390/medicina61020360 (PMC11857531; doi:10.3390/medicina61020360)
Supplement: Supplementary file 1 [file medicina-61-00360-s001.zip › medicina-3463429-supplementary.pdf]

Table S1. The first SRE after the initiation of BMA				
	Total (n = 328)	Denosumab n = 179 (54.6%)	Zoledronic Acid n = 149 (45.4%)	p-value
First post-BMA SRE				0.074
Yes	128 (39.0%)	62 (34.6%)	66 (44.3%)	
No	200 (61.0%)	117 (65.4%)	83 (55.7%)	
Type of first SRE				NA
RT to bone	70 (21.3%)	33 (18.4%)	37 (24.8%)	
Fracture	24 (7.3%)	11 (6.1%)	13 (8.7%)	
Surgery to bone	9 (2.7%)	5 (2.8%)	4 (2.7%)	
Cord compression	18 (5.5%)	10 (5.6%)	8 (5.4%)	
Hypercalcemia	7 (2.1%)	3 (1.7%)	4 (2.7%)	

SRE: skeletal-related events; BMA: bone-modifying agents; RT: radiotherapy; NA: not applicable
